# Recent Progress in the Identification of Aptamers Against Bacterial Origins and Their Diagnostic Applications

**DOI:** 10.3390/ijms21145074

**Published:** 2020-07-18

**Authors:** Nevina E. Trunzo, Ka Lok Hong

**Affiliations:** Department of Pharmaceutical Sciences, Nesbitt School of Pharmacy, Wilkes University, 84 W. South Street, Wilkes-Barre, PA 18766, USA; nevina.trunzo@wilkes.edu

**Keywords:** bacterial infection, bacterial diagnostic, aptamer, DNA, SELEX, molecular recognition element (MRE)

## Abstract

Aptamers have gained an increasing role as the molecular recognition element (MRE) in diagnostic assay development, since their first conception thirty years ago. The process to screen for nucleic acid-based binding elements (aptamers) was first described in 1990 by the Gold Laboratory. In the last three decades, many aptamers have been identified for a wide array of targets. In particular, the number of reports on investigating single-stranded DNA (ssDNA) aptamer applications in biosensing and diagnostic platforms have increased significantly in recent years. This review article summarizes the recent (2015 to 2020) progress of ssDNA aptamer research on bacteria, proteins, and lipids of bacterial origins that have implications for human infections. The basic process of aptamer selection, the principles of aptamer-based biosensors, and future perspectives will also be discussed.

## 1. Introduction

Bacterial infections in humans continue to be a significant challenge in both the community and hospital. In relation to bacterial infection is the increasing emergence of antibiotic resistance. The U.S. Centers for Disease Control and Prevention (CDC) reported more than 2.8 million cases of antibiotic-resistant infections were observed in the U.S. each year, and more than 35,000 cases had led to death in their latest antibiotic resistance threat report [1]. One of the solutions to decrease antibiotic resistance is through the timely and sensitive diagnosis of bacterial infections. This can help to guide antibiotics selection in infected patients, and thus reduce antibiotic resistance. Currently, the bacterial cultural test is a standard method to obtain a confirmative result of the bacterial species [2,3]. However, some bacteria genera, such as mycobacteria, are difficult to culture. In these cases, molecular diagnostics techniques, such as polymerase chain reaction (PCR), or enzyme-linked immunosorbent assay (ELISA), or other antibody-based assays, are used to facilitate the bacterial diagnosis [4,5].

In the last three decades, molecular recognition elements (MRE) that are alternatives to antibodies have been heavily investigated in the area of biosensing and diagnostics. One of the most promising MREs is nucleic acid-based binding elements due to high affinity and specificity. The first reported method of screening an RNA binding element was reported by the Gold Laboratory [6]. Shortly after, Szostak and Ellington reported a different RNA binding element and termed it aptamer [7]. Aptamers can bind to a variety of bacterial targets, including whole cells, polysaccharides, proteins, toxins, and even spores that are generated by bacterium [8]. Due to the versatile nature of aptamers, there has been an exponential increase in aptamer therapeutics and diagnostics research [9].

**Figure 1:**
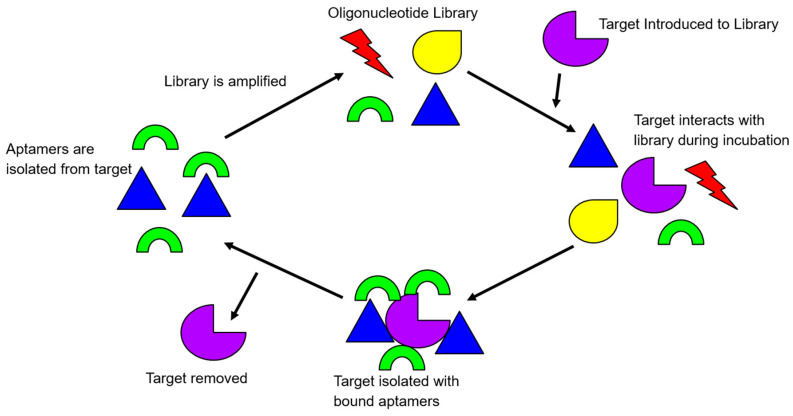
An illustration of the rudimentary principle of the Systematic Evolution of Ligands by Exponential Enrichment (SELEX) process. Target-bound library molecules are subjected to repeated cycles of incubation, partitioning, and amplification, to increase the library affinity for the target.

Because DNA molecules are generally more resistant to nuclease degradation than RNA molecules in vitro, single-stranded DNA (ssDNA) aptamers have received a higher amount of attention in their in vitro diagnostic and biosensing applications [10,11]. The process for identifying aptamers has been extensively reviewed previously [8,12,13,14]. In brief, aptamers are screened through the in vitro selection process, termed, systematic evolution of ligand by exponential enrichment, or commonly abbreviated as SELEX. It is an iterative process whereby a population of 10^13^ to 10^15^ different library molecules are subjected to repeated steps of incubation with the target molecule, separation, and amplification of bound library molecules. In the selection process of ssDNA aptamers, library molecules contain a random region flanked by two constant regions for PCR amplifications. The number of bases in the random region may vary, but usually, there is between 20–80 bases. The SELEX process has been modified to contain both positive (target), and negative (counter-targets) rounds, to direct the specificity of the library during the screening (Figure 1). 

To initiate a positive round of SELEX, the library of oligonucleotides is exposed to the target in question. After allowing the library to interact with the target, the target-bound complex is then washed of all non-binding library molecules. The library molecules that have effectively bound to the positive target are amplified via PCR. Subsequent rounds of SELEX start after the retrieval of the amplified library single-stranded DNA. Negative rounds of SELEX are usually integrated with the positive rounds of SELEX after several rounds of positive SELEX have been completed. This is to ensure the evolved library is robust enough to be screened by negative targets. A negative target is often similar to the target, “fooling” the less selective library oligonucleotides that have progressed through SELEX until that current point. The oligonucleotides that are less specific and bind to the negative target are, therefore, removed from the library. After completing approximately twelve to fifteen rounds of both positive and negative SELEX, a library can be deemed selective and specific, or whenever the number of target-bound library molecules plateaus [8].

Single-stranded DNA aptamers have many advantages over similar competitors. Firstly, they are more stable than RNA aptamers and still contain unique stem-loop structural variations [15]. Although antibodies have similar high affinity and specificity binding profiles when compared to single-stranded DNA aptamers, they are less thermally stable. Antibodies and other peptide-based binding elements are also irreversible after target recognition in diagnostics and biosensing applications. In addition, the process for selecting a single-stranded DNA aptamer can be performed independently of a living system, as opposed to antibodies. Lastly, single-stranded DNA aptamers can be chemically synthesized cost-effectively with minimal variation, adding yet another advantage to their usage [8].

Many variations can be made to single-stranded DNA aptamers to ease their biosensing and diagnostic applications, for example, the 3′ or 5′ ends of the aptamers may be modified to carry tags. These modifications allow the DNA aptamers to fluoresce or generate redox reactions upon target bindings [16]. This article focuses on reviewing recently (mid-2015 to early 2020) reported new single-stranded DNA aptamers and their usage within bacterial biosensing and diagnostics, that aim to facilitate the detection of bacterial infections in humans.

## 2. In Vitro Selection of ssDNA Aptamers

### 2.1. Overview of SELEX Methodology for Aptamer Specific for Bacterial Related Targets

The SELEX methodology has evolved over the last three decades from its original form, depending on the target for selection. Protein targets secreted from bacteria are commonly immobilized on magnetic beads or other solid platforms, such as 96-well plates for bound library molecule partitions. The libraries are often tagged with fluorescence dyes for monitoring library enrichment, such as the FluMag-SELEX [12]. Target-immobilization free techniques, such as the graphene oxide-based SELEX (GO-SELEX) are also useful in selection against small molecules, proteins, and viral particles [17,18,19]. However, when the targets are whole-cell bacteria, the Cell-SELEX, or Whole Cell-SELEX is the most commonly used technique. Frequently, the bacterial species is incubated with the library molecules, and followed by centrifugation partitioning of the bacterial pallet with the bound library [20]. Table 1 summarizes the recent aptamer selection reports on bacteria and bacterial related targets (Table 1). Studies discussing aptamer selection and their diagnostic applications will be summarized in a separate table.

### 2.2. Highlights on Recent ssDNA Aptamers Specific to Bacterial Origins 

#### 2.2.1. Staphylococcus Aureus and Its Related Proteins

*Staphylococcus aureus* is a common skin-dwelling bacteria. It acidifies the surface of the human body and can cause major infections when it enters into the internal human body. It is the cause of many infections, even including sepsis in severe cases. It is commonly seen as skin infections that cause swelling, warmth, and redness on the outer layers of skin. It has many components that make it toxic to humans in large doses, including secreted enterotoxins as well as cell surface proteins. Sepsis is often a very severe and life-threatening infection, and levels of bacteria need to be tested via blood samples. Several bacteria can cause this disease, but a very common culprit is *S. aureus*.

A study performed by Graziani et al. tested aptamers against multiple different bacteria that are known to cause sepsis [31]. The Kd estimated for the *Staphylococcus aureus* specific aptamer was 170.1 nM. The value was determined via SYBR Green real-time quantitative PCR. Another study identified aptamers specific to whole cells of *Staphylococcus aureus* after performing ten rounds of positive selection and three rounds with a negative target. The aptamers that were generated had a Kd between 34 and 128 nM [45]. 

Protein A is a common surface protein of *S. aureus*. Stoltenberg et al. used FluMag SELEX to generate a protein A binding aptamer. The reported Kd of the full length and truncated aptamers determined by surface plasmon resonance were in the micromolar range [43]. The group later utilized enzyme-linked oligonucleotide assays (ELONA) to obtain Kd values in the nanomolar range [46]. Noticeably, different binding experiment setups yield a wide range of affinity data. The same research group further studied this SELEX experiment, by analyzing the selected candidate pool with next-generation sequencing. They stated the originally obtained aptamer remained to be the best candidate for protein A [47].

*S. aureus* can also produce a group of enterotoxins that are responsible for staphylococcal food poisoning in many areas of the world. Several new aptamer selection studies were published for enterotoxin A, B, and C1. 

A novel method of SELEX termed the Staggered Target SELEX (ST-SELEX) was used to identify an aptamer for enterotoxin A (SEA) [48]. The reported dissociation constant value was 7.44 ± 0.6 nM after ten rounds of selection. ST-SELEX involved separate steps of classical SELEX, and a second SELEX aimed to reduce the library binding to enterotoxins that were homologous to enterotoxin A. The same research group also performed a different classical SELEX experiment to identify a different aptamer specific to SEA, with a reported Kd of 8.5 ± 0.91 nM [49].

Enterotoxin B, secreted by staphylococcal cells, generates a large reaction from T cells within the body, and can, therefore, cause Staphylococcal toxic shock syndrome. Wong et al. identified an aptamer with a Kd of 64 nM and tested its antagonistic effect in diminishing the toxin effect [44].

Enterotoxin C1 is another secretory enterotoxin of *S. aureus*. Huang et al. identified an aptamer with a Kd value of 65.14 ± 11.64 nM after twelve rounds of SELEX, using magnetic separation techniques [50]. The group also developed a graphene oxide-based assay to quantify enterotoxin C1 in food samples.

#### 2.2.2. *Pseudomonas aeruginosa*

*Pseudomonas aeruginosa* is also a bacteria that is commonly causing human infection in a variety of organs. This bacterium can infect the digestive tract, the urinary tract, the lungs, and other sites of elderly or immunocompromised individuals. Soundy et al. discovered an aptamer that bound to live *P. aeruginosa* cells, and with multiple reported dissociation constants in the low nanomolar range [36]. The candidates also demonstrated high specificity and were able to bind to both biofilm and planktonic growth of *P. aeruginosa*. The group reported the candidates lacked bacteriostatic and bactericidal activity, though it would be useful in diagnostic and biosensing applications. 

#### 2.2.3. Mycobacterium Species and Related Proteins

*Mycobacterium tuberculosis* is the causative bacteria for tuberculosis. While it is uncommon in developed countries, it remains a problem in many other areas of the world. Multiple aptamers have been developed that target strains of this bacterium or virulence factors for its havoc in humans. Mozioglu et al. isolated the H37Ra strain as the positive target in their SELEX study [33]. Both ultrafiltration and centrifugation methods were used to partition the bound and unbound library molecules. At the conclusion of the study, an aptamer with a Kd of 5.09 ± 1.43 nM was obtained. 

Zhang et al. selected an aptamer specific for the H37Rv strain *M. tuberculosis* with a Kd of 37 ± 4 nM [51]. The research group also developed a multichannel series piezoelectric quartz crystal sensor using the selected aptamer, single-walled carbon nanotube, and gold electrode, to achieve a detection limit of 100 CFU/mL in seventy minutes.

Aimaiti et al. identified an aptamer for the H37Rv strain, with a Kd of 12.02 nM after ten rounds of selection [52]. The group also developed a sandwich enzyme-linked immunosorbent assay using a combination of five aptamers for capturing and detecting the specific strain.

In addition to selecting aptamers specific for the whole cell *M. tuberculosis*, there has been an aptamer developed for a particular component of the bacterium, the mannose-capped lipoarabinomannan (ManLAM) on the outer surface. This lipoglycan serves a vital role in cell to cell communication. Tang et al. identified an aptamer specific for *T. tuberculosis* ManLam lipoglycan, with a dissociation constant of 668 ± 159 nM. The author also developed an enzyme-linked oligonucleotide assay (ELONA) to detect the ManLAM antigen in patient serum and sputum samples with active TB infections.

Sun et al. selected an aptamer that bound to ManLAM molecules of *Mycobacterium bovis*, or commonly termed bacillus Calmette–Guerin (BCG). The reported Kd of this aptamer was 8.59 ± 1.23 nM. The author stated the potential use of the aptamer as an immune enhancer for BCG. 

*M. tuberculosis* is also capable of secreting proteins to further the course of human infection, such as the MPT64 secretory protein. Following ten cycles of SELEX, an aptamer with a Kd of 8.92 nM was identified [32]. The identified aptamer was also incorporated into an ELONA assay for the specific and sensitive detection of MPT64 in clinical sputum samples.

Another study was conducted to detect the Mycobacterium Ag85A secretory complex. An aptamer was selected using magnetic beads [53]. The reported Kd was 63 nM. The author also developed a rapid fluorescent assay with the aptamer and graphene oxide. The reported limit of detection was 1.5 nM.

#### 2.2.4. *Escherichia coli*

Pathogenic strains of *Escherichia coli* continue to generate interest in many aptamer research groups. 

Marton et al. identified four aptamers specific to a laboratory strain of *E. coli* [26]. The reported Kd values ranged from 11.97 nM to 161 nM. The author stated that one of the twelve aptamer candidates was highly specific to *E. coli* and was able to bind to meningitis/sepsis associated *E. coli* (MNEC). They indicated the potential for the candidate aptamer to be used in diagnosing MNEC associated infections.

Masoum et al. isolated an aptamer specific for *E. coli* O157:H7, a pathogenic strain that causes foodborne illnesses in humans [24]. Following nine rounds of positive selection and a final tenth round of negative selection, the author reported a highly specific aptamer with a Kd of 107.6 ± 67.8 pM. The author stated the potential for using it in diagnostic and biosensing of bacteria in food.

Zou et al. further examined the possibility of identifying an aptamer specific for different stages of the O157:H7 strain [25]. The reported aptamer had a Kd of 9.04 ± 2.80 nM. The author concluded lipopolysaccharide (LPS) could potentially be the molecular target of the aptamer.

Yu et al. again selected an aptamer for the O157:H7 strain *E. coli* [54]. After nineteen total rounds of SELEX with six rounds of negative selection, the reported aptamer had a dissociation constant of 10.3 nM. The author also developed a quartz crystal microbalance aptasensor to detect the strain. The limit of detection was 1.46 × 10^3^ CFU/mL.

A similar strain, O78:K80:H11, was also chosen for the aptamer selection experiment. Kaur et al. utilized a total of twelve rounds of SELEX, with rounds one through five consisting of positive selections and rounds six through eleven consisting of negative selections [55]. The reported dissociation constant was approximately 14 nM. The aptamer was also incorporated into a label-free impedimetric aptasensor for the sensitive detection of the strain in liquid samples. The reported limit of detection was about 10 CFU/mL.

Another *E. coli* strain, DH5α, was examined as a target by Renders et al. [27]. The author obtained a sensitive aptamer, with a Kd of 27.4 ± 18.7 nM, after performing twelve rounds of whole cell SELEX.

Song et al. took a different approach to identify aptamers that were specific to not only *E. coli*, but to other disease-causing gram-positive and gram-negative bacteria [38]. This includes *Enterobacter aerogenes*, *Klebsiella pneumoniae*, *Citrobacter freundii*, *Bacillus subtilis*, and *Staphylococcus epidermidis*. This was performed via a novel SELEX, termed the sequential toggle cell SELEX (STC-SELEX). In this SELEX methodology, one of the six bacterial groups was incubated with the library at once. After the library was exposed to one bacterial group, it was allowed to be exposed to the next, and so on. The isolated aptamers had reported dissociation constants in the low nanomolar range. They were also only specific to the bacteria species that were included in the selection.

#### 2.2.5. Streptococcus Species

*Streptococcus pyogenes* can overgrow in the throat, particularly on the tonsils. These symptoms are accompanied by soreness in the throat, as well as a fever, and is commonly called strep throat. Hamula et al. subjected two sets of libraries to look for aptamers specific for *S. pyogenes*. Several aptamers were selected, with dissociation constants ranging from 7 nM to 71 nM [28]. 

*Streptococcus mutans* is an oral infective agent in humans. A specific aptamer with a Kd of 69.45 ± 38.53 nM was identified after nine rounds of whole cell SELEX. This aptamer was highly specific to cariogenic clinical *S. mutans (Streptococcus mutans)* strains [30]. 

Group A streptococcus expresses serotype M3 on its surface. This surface protein generates a more lethal infection for human hosts, and therefore this is a more severe pathogenic strain of the species. Using this specific strain of streptococcus, Alfavian et al. identified an aptamer with a Kd value of 7.47 ± 1.72 pM [29].

*Streptococcus pneumoniae* can cause fever and chest congestion, as well as airway irritation, swelling, and cough. The associated infection can be fatal in elderly and immunocompromised patients. Bayrac et al. reported several aptamers capable of binding specifically to this species of streptococcus [56]. After ten rounds of whole cell SELEX, three aptamers were selected with the following Kd values, Lyd−1 = 844.7 + 123.6 nM Lyd−2 = 1984.8 + 347.5 nM Lyd−3 = 661.8 + 111.3 nM. A graphene oxide based fluorescent assay was developed using the Lyd−3 aptamer. The reported limit of detection was 15 CFU/mL.

#### 2.2.6. *Bacillus Anthracis* Virulence Factors

Anthrax, although rare, can have dire consequences of infection. Biondi et al. reported using an artificially expanded genetic information system (AEGIS) to perform a SELEX experiment with two additional artificial nucleotides [40]. The author identified an aptamer with high affinity and specificity toward the protective antigen (PA63) of *Bacillus anthracis*. After the completion of fourteen rounds of selection, the author reported a specific aptamer with a dissociation constant of 35 nM. 

Lahousse et al. identified an aptamer against a different virulence factor, the lethal factor (LF) of *B. anthracis* (*Bacillus anthracis*) [39]. The reported Kd was 11 ± 2.7 nM. The aptamer selected also had a potent inhibition on *B. anthracis*. The author expressed the potential of using the aptamer as a gateway to effective treatments in the future.

#### 2.2.7. Vibrio Species

Vibrio species bacteria reside mostly in oceans and can cause dangerous food borne illnesses in humans. *Vibrio parahemolyticus* is a common foodborne pathogen. Song et al. utilized a modified graphene oxide SELEX, coupled with isothermal rolling circle amplification to identify an aptamer specific for *V. parahemolyticus (Vibrio parahemolyticus)* [22]. The best aptamer had a reported Kd of 10.3 ± 2.5 nM. Specificity of this aptamer was tested via flow cytometry. Both negative and positive rounds were utilized to identify the aptamers, with only six rounds of selection being performed total. 

*Vibrio alginolyticus* is capable of infecting both marine lives as well as humans. Yu et al. identified two aptamers specific for the species [21]. The reported dissociation constants were 14.31 ± 4.26 nM and 90 ± 13.51 nM. The author noted that the aptamers did not have cytotoxic effects both in vitro and in vivo and could be used as a probe for sensitive detection of *V. alginolyticus*.

Yan et al. selected an aptamer specific for *Vibrio vulnificus* [23]. After ten positive and three negative rounds of selection, an aptamer was identified. The reported Kd was 26.8 + 5.3 nM. The author stated the aptamer could specifically detect the pathogen at a range between 8 to 2.0 × 10^8^ CFU/mL.

#### 2.2.8. *Helicobacter pylori*

Another bacterial species that normally resides within humans is *Helicobacter pylori*. It usually resides within the stomach of humans. It can cause infection and symptoms in humans when there is extreme growth. Yan et al. selected a specific aptamer against the recombinant surface protein of *H. pylori* [42]. The reported dissociation constant was 26.48 ± 5.72 nM. Although the aptamer was selected against a protein target, it showed specific binding to *H. pylori* cells.

## 3. Diagnostic and Biosensing Applications of ssDNA Aptamer for Bacterial Infection

### 3.1. Overview of Common Detection Principles

In general, the majority of the investigational ssDNA aptamer-based diagnostic and biosensing platforms can be divided into two broad categories, (1) optical and (2) electrical/electrochemical. The former category includes fluorescence, chemiluminescence, and colorimetric and surface plasmon resonance detections. The latter category most commonly includes amperometric and impedimetric sensors.

**Figure 2:**
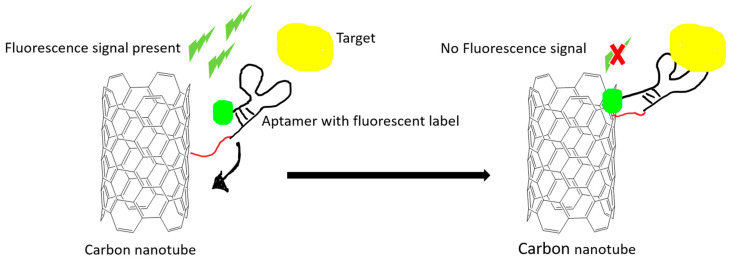
An example illustration of how carbon nanomaterials are used as a fluorescence quencher to turn off the signal. The fluorescence signal is quenched after the target-induced conformational change in the aptamer. The quenching effect is due to the Förster resonance energy transfer (FRET) effect.

Optical proof-of-principle detections are relatively easy to develop and operate. The aptamer is often conjugated with various fluorescence molecules, such as 6-carboxy-fluorescein (FAM), cyanine, and quantum dots. Using quantum dots has the advantage of overcoming photobleaching effects that are common in fluorescent dyes. Förster or fluorescence resonance energy transfer (FRET) is a special phenomenon that relies on energy transfer between two light sensitive molecules in a very short distance. Carbon nanomaterials have been utilized as a signal quencher in FRET-based aptamer sensors (Figure 2).

**Figure 3:**
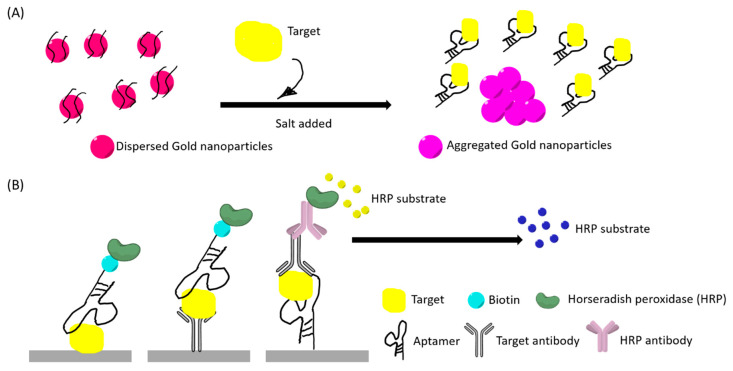
(**A**) An example illustration of a “red to purple” colorimetric sensor using gold nanoparticles (AuNPs). The left had side illustrates the initial stage where AuNPs are dispersed by aptamer-coating, thus having a red appearance. AuNPs aggregated after target bind to the aptamer and salt addition. (**B**) An example illustration of aptamer enzyme-linked sorbent assays. Primary antibody for the target and secondary antibody can be used in different sandwich assays. The color changes in both (**A**) and (**B**) can be observed with naked eyes and quantified by optical instruments.

The colorimetric detection method often utilizes gold nanoparticles (AuNP) for rapid detection. This method is generally low-cost and user-friendly. Salt addition can induce changes in the aggregated size of the nanoparticles and lead to a red to purple color shift that is visible to the naked eye (Figure 3A). These factors have led to many studies developing aptamer-based colorimetric assays. Aptamers can also be incorporated into peroxidase enzyme-linked assays, capable of generating color changes that are readable as absorbance values (Figure 3B). 

**Figure 4:**
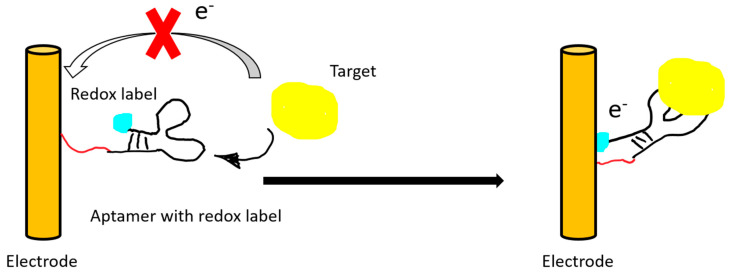
An illustration of a “signal on” electrochemical sensor. The electrical signal of the redox label is recorded after target binding and subsequent conformational change of the aptamer.

Electric or electrochemical aptasensors can be label-free or labeled. In a labeled aptasensor, a redox tag on the aptamer is needed for transferring the electron upon target binding. The electron transfer can then be translated into an amperometric or an impedimetric reading (Figure 4). 

**Figure 5:**
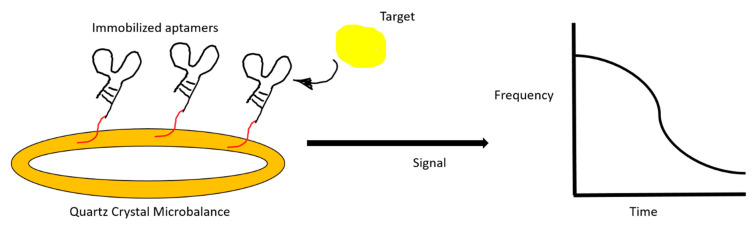
An illustration of a quartz crystal microbalance aptasensor. The change in resonance frequency after target binding is registered on a monitor.

A label-free aptasensor can utilize mass-sensitive techniques, such as quartz crystal microbalance (QCM) and micromechanical cantilever. These piezoelectric biosensors can generate a resonance frequency change after target binding (Figure 5).

**Figure 6:**
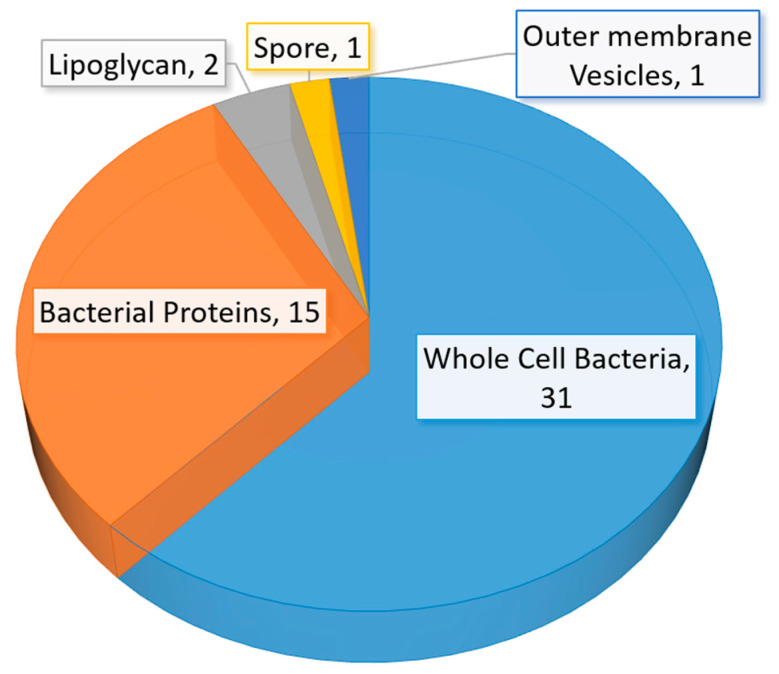
A graphical representation of the number of newly identified aptamers against bacteria and related factors from mid-2015 to early 2020. This pie chart includes aptamers from both Table 1 and Table 2.

**Figure 7:**
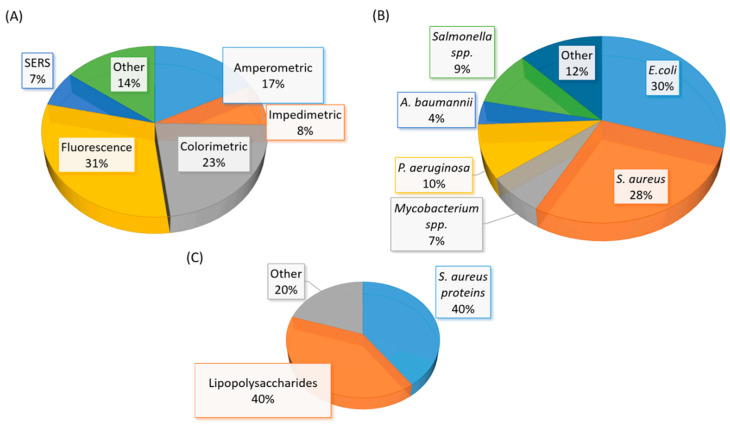
(**A**) A graphical representation of the type of aptasensor summarized in Table 3. (**B**) A graphical representation of the whole-cell bacterial species detected in Table 3. (**C**) A graphical representation of the types of bacterial component detected in Table 3. The total number of articles summarized in Table 3 is fifty-eight. Note: Eight studies reported the detection of more than one bacterial species (spp.)

A significant amount of research related to aptamer selections and diagnostic applications were published between mid-2015 to early 2020. The number of studies in aptamer-based diagnostic and biosensor development is also beyond the capability of a full discussion in this review. Thus, we have elected to discuss selected studies based on the area or location of the infection in humans. The summary of recent (mid-2015 to early 2020) bacterial-related aptamer selection and diagnostic application studies are listed in Table 2 (Table 2). The total number of newly identified ssDNA aptamers for bacteria and bacterial components are illustrated in a pie chart (Figure 6). Likewise, bacterial diagnostic sensor studies in this period are summarized in Table 3. Aptasensors designed solely for pathogen detection in food and drink samples are excluded in this table (Table 3). The types of aptasensor and the categories of the detected target are illustrated in Figure 7.

### 3.2. Highlights of ssDNA Aptamer-Based Diagnostics for Bacterial Skin Infections

Bacterial skin infections in the hospital are extremely prevalent and can be easily transmissible from patient to patient, or even patients to hospital staff. A common culprit of skin infections is *Staphylococcus aureus*. This bacteria is the most common cause of skin infections, as it is normally present on the skin, but if it overgrows, it can cause infections that can even cause sepsis [146]. There was a reported aptasensor that could detect *S. aureus* very sensitively at 1.5 CFU/mL [79]. This aptasensor was based upon the surface-enhanced Raman scattering principle or SERS. The aptamer changed its configuration to optimally bind to the *S. aureus* bacteria, and then this complex acted as a template for gold nanoparticles to bind to. An infrared light was utilized to beam through the sample. A unique shift in vibrational energy was generated in the presence of gold-bound aptamer and bacteria complex, therefore confirming the presence of the bacteria.

*S. aureus* infection can be so detrimental and difficult to treat due to a commonly secreted enterotoxin [146]. An aptasensor specific for staphylococcus enterotoxin B was created utilizing the multicolor time-resolved fluorescence method [139]. It utilized graphene oxide as a resonance acceptor. The presence of the target bacteria caused a signal quench, thus turning off the sensor. The reported toxin concentration detection limit was at 1 ng/mL.

Methicillin-resistant *Staphylococcus aureus* (MRSA) infections in humans are often resistant to several antibiotic treatments and can require a longer course of medication [147]. A study reported detecting this bacterium in a microfluidic system and achieved a limit of detection at 450 CFU/mL [117]. In order to recognize this bacteria target, a nitrocellulose membrane attached to a glass slide was coated with the aptamer, and the bacteria were then incubated on the membrane. Unbound materials were washed away, and the captured bacteria that remained on the membrane received another round of aptamer exposure and additional rounds of washing for unbound materials. The secondary aptamer was labeled with biotin, and after washing, the tetramethylbenzidine substrate reacted with the horseradish peroxidase. The change in color was observed, and the absorbance level was measured to approximate the bacterial concentration [117].

### 3.3. Highlights of ssDNA Aptamer-Based Diagnostics for Bacterial Respiratory System Infections

It is generally more difficult to infect the respiratory tract, because of an array of defense mechanisms present along the tract. However, bacteria such as *Mycobacterium tuberculosis* can cause lung infection in humans. The thick sputum can harbor the bacteria and related virulence factors [148]. An ultrasensitive electrochemical aptasensor was developed recently [73]. It utilized gold nanoparticles decorated fullerene-doped polyaniline as the redox label to generate an amplified electrochemical signal upon binding to the *M. tuberculosis* antigen, MPT64. The reported limit of detection was at 20 fg/mL. It also demonstrated specificity and sensitivity for MPT64 in clinical serum samples [73].

*Acinetobacter baumanii* is another prevalent bacterium that continues to cause respiratory tract infections. It can develop and frequently be transmitted between hospitalized patients. This bacterium can originate in the skin, soil, water, or food, and can rapidly be transferred to generate multidrug-resistant strains [149]. An enzyme-linked aptamer sorbent assay (ELASA) was utilized to measure the fluorescence signal upon aptamer binding to the bacteria [62]. The assay showed good specificity over other commonly observed bacteria in healthcare settings, such as *P. aeruginosa*, and *E. coli*. The sandwich ELASA assay demonstrated a sensitivity of 95.47% on clinical isolates [62].

Perhaps one of the most commonly seen respiratory infection is caused by *Streptococcus pneumoniae*. It mainly infects people with compromised lung function or the elderly. It has proven to have a high rate of mortality [150]. A fluorescent assay was developed using graphene oxide. The aptamers showed selectivity against *E. coli*, *S. pyogenes*, and *S. aureus*. The limit of detection for this apparatus was as low as 15 CFU/mL [56].

### 3.4. Highlights of ssDNA Aptamer-Based Diagnostics for Other Infections

In addition to the infection types listed above, bacteria continue to cause a plethora of symptoms in human hosts. Not simply limited to one organ system, these bacteria can cause a unique array of symptoms, and this can, therefore, make them even more deadly. 

For example, *Pseudomonas aeruginosa* is a bacteria that can be found in soil, food, and water, and can cause a wide array of human symptoms like urinary tract infections, pneumonia, lung disease, and cystic fibrosis [151]. Because of its broad scope of symptoms, this bacterium is particularly troublesome for their human hosts and often results in more severe infections. An aptasensor was created for this bacterium that was unique because it utilized two different strategies, Surface-enhanced Raman spectroscopy (SERS), and colorimetry [113]. The aptamer was bound to gold nanoparticles, with the larger gold nanoparticles and aptamer complexes acting for colorimetry, and the smaller complexes acting as signalers for SERS. When the bacteria were not present, the two complexes bound together, and no signal was transmitted. This aptasensor could detect as low as 20 CFU/mL of the bacteria in solution [113].

Another non-traditional bacteria that can infect humans is *Bacillus anthracis*. Its spores spread its ability to transmit infection, and therefore its spores were the target of sensitive detection. This bacterium is known for its ability to be a biological weapon and can cause general signs of infection, such as fever, shortness of breath, sweating, nausea, and vomiting [152]. These spores were known as *B. cereus* spores, and the limit of detection of a reported impedimetric aptasensor was at 3 × 10^3^ CFU/mL [135]. The aptamer was first immobilized on gold electrodes, with a redox label, Fe(CN_6_), immersed in the environment. When the target was introduced into thesystem, the Fe(CN_6_) was displaced from the aptamer, and the decrease of resistance was measured [135].

An additional bacteria species that can uniquely cause disease in humans is *Vibrio alginolyticus*. It is resistant to salt and can thrive in oceans. It can cause infections in open wounds and ears in humans [153]. Zhao et al. described a method of sensing this bacteria using magnetic beads [141]. With a “capture DNA” fixed on the surface of the magnetic beads, the aptamer combined with the complex and bound to the target when it was present. The electrode sensed the aptamer detached from the beads as a result of target-binding. It measured the electrostatic interactions with a limit of detection of 10 CFU/mL [141].

## 4. Conclusions and Future Perspective

Pertinent and emerging new diseases will require precise and rapid diagnosis to facilitate proper treatment. Since the first description of the aptamer technology three decades ago, aptamers have shown to be very adaptive in binding to a wide range of targets. This feature has allowed them to be investigated as the recognition and binding element in diagnostic and biosensing applications. A major advantage of aptamer technologies resides within the selection process. It can identify new binding elements for previously unknown sites or motifs on the bacterium. Newly discovered aptamers can help to map novel binding sites that could have therapeutic potentials, in addition to diagnostic implications. Aptamers specific for bacterial virulence factors have shown to demonstrate neutralization abilities [44,49]. These aptamers could have the potential to be applied as a combined therapeutic and diagnostic agent, or a theranostic agent.

Although a vast amount of research has been conducted in the aptamer field in recent years, there is still a lack of commercially available aptasensors for infectious disease diagnosis. One of the challenges that is unique in detecting living organisms lies in the aptamer specificity for the pathogenic strains of the bacterium. Many aptamer selection studies had utilized bacteria from laboratory cultures. These laboratory strains may be different than clinical isolates from patients, and thus could lead to potential false negatives or false positives. In addition to factors related to the aptamer binding profile, sensor setup and detection principles are also crucial to sensitive and specific detection of the target virulence factor or bacterium. Clinical samples such as whole blood, plasma, saliva, and sputum are complex biological matrices. Enzyme degradation, non-specific binding to serum proteins, and non-pathogenic normal flora are all likely to interfere with the binding aptamers. The aptamer selection process must be designed to overcome potential interferences from components in these biological fluid samples. The amount of sample preparation is also a factor that can dictate the type of sensing principle in aptasensors. Whole blood samples are not ideal for colorimetric sensing because of the natural color of hemoglobin. Serum samples containing albumin can also interfere with fluorescence signals. In comparison, electrochemical aptasensors have an advantage in overcoming these optical interferences.

Recently, Wan et al. reported using an aptamer cocktail consisting of multiple aptamers with varying motifs, to detect *Mycoplasma* in cell culture [154]. This strategy could be useful in the detection of bacteria in clinical samples. The selection process often generates multiple aptamer candidates. Additional motif analysis and experiments could be performed in most bacterial whole-cell SELEX, as there is a high potential for multiple interaction sites between the relatively large bacterial cell and the aptamer library molecules.

It is evident that aptamer-based biosensors or aptasensors require a multidisciplinary collaboration for success. The biologists, chemists, and engineers all play a crucial role in integrating the aptamer technology. As we are moving into the fourth decade of aptamer research, it is reasonable to believe that the limitless future of aptamers remain to be discovered.

## Figures and Tables

**Table 1:** Summary table of new single-stranded DNA (ssDNA) aptamers selected against bacteria and bacterial related targets.

Target	SELEX Method	Equilibrium Dissociation Constant	Class of Molecule	Reference
*Vibrio alginolyticus*	Whole cell	14.31 ± 4.26 nM (VA2) and 90 ± 13.51 nM (VA8)	Bacterial cell	[21]
*Vibrio parahemolyticus*	Whole cell coupled with graphene oxide and isothermal amplification	10.3 ± 2.5 nM	Bacterial cell	[22]
*Vibrio Vulnificus*	Whole cell	26.8 ± 5.3 nM	Bacterial cell	[23]
*Escherichia coli*	Whole cell	107.6 ± 67.8 pM	Bacterial cell	[24]
Different stages of *E. coli* (*Escherichia coli*) O157:H7	Whole cell	9.04 ± 2.80 nM	Bacterial cell	[25]
*Escherichia coli*	Whole cell	Four aptamers: range from 11.97 to 161 nM	Bacterial cell	[26]
*E. coli* DH5 alpha	Whole cell	27.4 ± 18.7 nM	Bacterial cell	[27]
*Streptococcus pyogenes* (M type)	Flow cytometry assisted	Low nanomolar range	Bacterial cell	[28]
M-type 11 *Streptococcus pyogenes*	Whole cell	7 ± 1 nM	Bacterial cell	[28]
Group A *Streptococcus* Serotype M3	Whole cell	7.47 ± 1.72 pM	Bacterial cell	[29]
*Streptococcus mutans*	Subtractive (Whole cell) SELEX	69.45 ± 38.53 nM	Bacterial cell	[30]
Sepsis Bacterium (*A. baumanii (Acinetobacter baumanii), L. monocytogenes (Listeria monocytogenes), E. coli, S. aureus (Staphylococcus aureus), K. pneumonia (Klebsiella pneumonia)*)	Whole cell	To each bacteria in order, Antibac 1 (268.5 ± 54.34, 51.74 ± 11.75, 31.82 ± 4.38, 170.10 ± 32.13, 256.10 ± 47.89) nM; Antibac 2 (71.92 ± 9.74, 54.19 ± 12.09, 62.43 ± 11.97, 194.90 ± 38.55, 195.90 ± 42.91) nM	Bacterial cell	[31]
MPT 64 (*Mycobacterium tuberculosis* secretory protein)	Nitrocellulose membrane	8.92 nM	Protein	[32]
*Mycobacterium Tuberculosis* H37RA	Whole cell	5.09 ± 1.43 nM	Bacterial cell	[33]
Mannose-Capped Lipoarabinomannan of Bacillus Calmette–Guérin	96-well plate	8.59 ± 1.23 nM	Lipoglycan	[34]
Mannose-Capped Lipoarabinomannan of *Mycobacterium tuberculosis*	96-well plate	668 ± 159 nM	Lipoglycan	[35]
*Pseudomonas aeruginosa*	Whole cell	Low nanomolar range	Bacterial cell	[36]
*Salmonella Typhimurium*	Magnetic assisted Cell SELEX	6.33 ± 0.58 nM	Bacterial cell	[37]
*Escherichia coli, Enterobacter aerogenes, Klebsiella pneumoniae, Citrobacter freundii, Bacillus subtilis, and Staphylococcus epidermidis*	Sequential toggle cell-SELEX	9.22 nM to 38.5 nM	Bacterial cell	[38]
Lethal factor (*Bacillus anthracis*)	Electrophoretic mobility shift assay	11 ± 2.7 nM	Protein	[39]
Protective antigen (*Bacillus anthracis*)	Magnetic beads	35 nM	Protein	[40]
*Bacillus cereus* spores	Unpublished	5.2 ± 52.4 nM	Bacterial spores	[41]
*H. pylori (Helicobacter pylori)* surface recombinant antigen	96-well plate	26.48 ± 5.72 nM	Protein	[42]
Protein A (*S. aureus*)	FluMag-SELEX	Low to submicromolar range	Protein	[43]
Staphylococcal enterotoxin B	Magnetic beads	64 nM	Protein	[44]

**Table 2:** Summary table of new ssDNA aptamers selected against bacteria and bacterial related targets and their diagnostic and biosensing applications.

Target	SELEX Method	Kd	Detection Method	LOD	Class of Molecule	Reference
*Shigella sonnei*	whole cell	SS−3: 39.32 ± 5.02 nM and SS4: 15.89 ± 1.77 nM	Fluorescence	10^3^ cells per mL	Bacterial cell	[57]
*M. tuberculosis*	whole cell	37 ± 4 nM	Piezoelectric quartz crystal	100 CFU/mL	Bacterial cell	[51]
*Salmonella Enteritidis*	whole cell	crn−1: 0.971 µM and crn−2: 0.309 µM	Colorimetric	10^3^ CFU/mL	Bacterial cell	[58]
*Neisseria meningitidis*	whole cell	K3: 28.3 ± 8.9 pM K4: 39.1 ± 8.6 pM	Fluorescence	200 CFU/mL (infected) 100 CFU/mL (artificially infected)	Bacterial cell	[59]
*E. coli* O78:K80:H11 strain	whole cell	14 nM	Label free impedimetric	10 CFU/mL	Bacterial cell	[55]
*Salmonella enteritidis*	whole cell	80 nM	Fluorescence	25 CFU/mL	Bacteria cell	[60]
*Salmonella enterica* ser. Typhimurium	whole cell	0.00214 ± 0.00312 µM	Fluorescence	2 × 10^1^ to 2 × 10^5^ CFU/mL	Bacterial cell	[61]
Staphylococcal enterotoxin A (SEA)	whole cell	8.5 ± 0.91 nM	Surface plasmon resonance	5 ng/mL	Protein	[49]
*Acinetobacter baumanii*	whole cell	Aci49: 7.547 ± 1.353 pM Aci55: 10.70 ± 2.561 pM	Colorimetric (ELASA)	10^3^ CFU/mL	Bacterial cell	[62]
Glutamate dehydrogenase (*Clostridium difficile*)	Magnetic beads	anti-GDH1: 3.1 ± 1.2 nM anti-GDH3: 5.6 ± 2.4 nM anti-GDH7: 4.6 ± 1.6 nM	FRET	1 nM	Protein	[63]
*Streptococcus pneumonia*	Whole cell	Lyd−1: 844.7 ± 123.6 nM Lyd−2: 1984.8 ± 347.5 nM Lyd−3: 661.8 ± 111.3 nM	GO based fluorescent assay	15 CFU/mL	Protein	[56]
Staphylococcal enterotoxin A (SEA)	Staggered target SELEX	7.44 + 0.6 nM	Apta-qPCR	146.67 fM	Bacterial cell	[48]
*E. coli* O157:H7	Whole cell	10.30 nM	Quartz crystal microbalance	1.46 × 10^3^ CFU/mL	Bacterial cell	[54]
Cholera Toxin	Semi-automated	23.2 - 56 nM	Sandwich enzyme linked aptamer assay	2.1 ng/mL (binding buffer) 2.4 ng/mL (tap water)	Protein	[64]
*M. tuberculosis* H37Rv strain	Whole cell	12.02 nM	Sandwich ELISA assay	1 × 10^3^ CFU/mL	Bacterial cell	[52]
*Staphylococcus aureus*	Whole cell	34 to 128 nM	Colorimetric	10^2^ CFU/mL	Bacterial cell	[45]
*Staphylococcus aureus* enterotoxin C1	Whole cell	65.14 ± 11.64 nM	Fluorescence	6 ng/mL	Protein	[50]
*Salmonella enterica* serovar typhimurium	Whole cell	SAL28: 195 + 46 nM SAL 11: 184 + 43 nM SAL 26: 123 + 23 nM	Fluorescence	10^3^ CFU/mL	Bacterial cell	[65]
*Listeria monocytogenes*	Whole cell	LMCA2: 2.01 × 10^−12^ M LMCA 26: 1.56 × 10^−10^ M	Fluorescence	20 CFU/mL	Bacterial cell	[66]
*Mycobacterium tuberculosis* Ag85A,	Magnetic beads	63 nM	GO based fluorescent assay	1.5 nM	Protein	[53]
*Pseudomonas aeruginosa* exotoxin A	Magnetic beads	4.2 to 4.5 µM	Sandwich aptamer modified ELISA assay	100 nM	Protein	[67]
*Vibro fischeri*	Whole cell	VFCA−02: 1.28 × 10^−10^ M VFCA−03: 25 × 10^−9^ M	Colorimetric	4 × 10^1^ CFU/mL	Bacterial cell	[68]
Gram-negative bacterial outer membrane vesicles	Toggle-cell-SELEX	20.36 to 59.70 nM	Enzyme-linked aptamer assay (ELAA)	25 ng/mL	Outer membrane vesicles	[69]
Staphylococcal enterotoxin B	Affinity chromatography	2.3 × 10^−11^ M	Enzyme-linked aptamer assay (ELAA)	5 ng	Protein	[70]
Penicillin binding proteins	X aptamer selection kit protocol	S3,15 nM S1 30 nM	Optical Colorimetric	20 nM	Protein	[71]

**Table 3:** Summary table of new ssDNA aptamer-based biosensors intended for the diagnostic of bacterial infection in humans.

Target	Detection Method	Limit of Detection	Class of Molecule	Reference
*Tuberculosis Meningitis* antigens	Electrochemical Amperometric	10 pg	Protein	[72]
*Mycobacterium tuberculosis* MPT64 antigen	Electrochemical Amperometric	20 fg/mL	Protein	[73]
*Mycobacterium tuberculosis* MPT64 antigen	Electrochemical Impedimetric	81 pM	Protein	[74]
*Mycobacterium tuberculosis* HspX antigen	Optical Aptamer linked immobilized sorbent assay Electrochemical Amperometric	~13 pM	Protein	[75]
*Mycobacterium tuberculosis* strain H37Rv	Electrochemical piezoelectric quartz crystal	100 CFU/mL	Whole cell	[76]
*Staphylococcus aureus*	Fluorescence	682 cells	Whole cell	[77]
*Staphylococcus aureus*	Optical Colorimetric	16 CFU/mL	Whole cell	[78]
*Staphylococcus aureus*	Surface-enhanced Raman scattering (SERS)	1.5 CFU/mL	Whole cell	[79]
*Staphylococcus aureus*	Surface-enhanced Raman scattering (SERS)	3 cells/mL	Whole cell	[80]
*Staphylococcus aureus*	Electrical Piezoelectric quartz crystal	41 CFU/mL	Whole cell	[81]
*Staphylococcus aureus*	Fluorescence	93–270 CFU/mL	Whole cell	[82]
*Staphylococcus aureus*	Electrochemical Impedimetric	1 CFU/mL	Whole cell	[83]
*Staphylococcus aureus*	Optical Colorimetric	20 CFU/mL	Whole cell	[84]
*Staphylococcus aureus*	Pressure Readout Using Aptamer-Coated Magnetic CuFe_2_O_4_ and Vancomycin-Capped Platinum Nanoparticles	1 CFU/mL	Whole cell	[85]
*Staphylococcus aureus*	Colorimetric Absorbance	81 CFU/mL	Whole cell	[86]
*Staphylococcus aureus*	Optical Chemiluminescence	5 CFU/mL	Whole cell	[87]
*Staphylococcus aureus*	Surface-enhanced Raman scattering (SERS)	10 cells/mL	Whole cell	[88]
*Staphylococcus aureus*	Fluorescence	1.7 CFU/mL	Whole cell	[89]
methicillin-resistant *Staphylococcus aureus* (MRSA)	Fluorescence	2.63 × 10^3^ (PBS) 1.38 × 10^3^ (spiked nasal swab)	Whole cell	[90]
methicillin-resistant *Staphylococcus aureus* (MRSA)	Optical Colorimetric	Not mentioned	Whole cell	[91]
*E. coli and S. aureus*	Optical Colorimetric	100 CFU/mL	Whole cell	[92]
*E. coli and S. aureus*	FRET	3 CFU/mL	Whole cell	[93]
*E. coli and S. aureus*	Electrical Capacitance sensor	10 CFU/mL	Whole cell	[94]
*E. coli and S. aureus*	Electrical Conductometric	2.3 × 10^4^ CFU·mL−1 and 4.0 × 10^3^ CFU/mL for *E. coli* and *S. aureus*	Whole cell	[95]
*Escherichia coli* O157:H7	Optical Colorimetric Lateral flow assays	10 CFU/mL	Whole cell	[96]
*Escherichia coli* O157:H7	Optical Chemiluminescence	4.5 × 10^3^ CFU/mL	Whole cell	[97]
*Escherichia coli* O157:H7	Electrochemical Impedimetric	2 CFU/mL	Whole cell	[98]
*Escherichia coli* O157:H7	Electrochemical Amperometric	32 CFU/mL	Whole cell	[99]
*Escherichia coli* O157:H7	Photoelectrochemical aptasensor using CdS Quantum dots/Au nanoparticles/ZnO Nanowire Array	1.125 CFU/mL	Whole cell	[100]
*Escherichia coli* (ETEC) K88	Fluorescence	10^2^ CFU/mL	Whole cell	[101]
*Escherichia coli* K88	Optical Colorimetric	1.35 × 10^2^ CFU/mL	Whole cell	[102]
*Escherichia coli*	Optical Electrochemiluminescene	0.17 CFU/mL	Whole cell	[103]
*Escherichia coli*	Electrochemical Photocurrent	0.66 CFU/mL	Whole cell	[104]
*Escherichia coli*	Electrochemical Amperometric	8 CFU/mL	Whole cell	[105]
*Escherichia coli*	Electrochemical Amperometric	100 CFU/mL	bacteria	[106]
*Escherichia coli*	Non-Faradaic Impedance Biosensor	9 CFU/mL	Whole cell	[107]
Antibiotic resistant *E. coli*	Fluorescence	60 CFU/mL	Whole cell	[108]
*E. coli and Salmonella*	Fluorescence	100 CFU/mL	Whole cell	[109]
*Salmonella typhirium and Pseudomonas aeruginosa*	Optical Localized Surface Plasmon Resonance	30 CFU/mL	Whole cell	[110]
*Pseudomonas aeruginosa*	Optical Colorimetric Electrochemical Amperometric	60 CFU/mL	Whole cell	[111]
*Pseudomonas aeruginosa*	Optical Fluorescence	1 CFU/mL	Whole cell	[112]
*Pseudomonas aeruginosa*	Surface-enhanced Raman scattering Optical Colorimetric	20 CFU/mL	Whole cell	[113]
*Pseudomonas aeruginosa* strain PAO1	Optical Localized Surface Plasmon Resonance sensor	a single bacterium	Whole cell	[114]
*Pseudomonas aeruginosa*	Electrochemical Amperometric	2 CFU/mL	bacteria	[115]
*Pseudomonas aeruginosa* and methicillin-resistant *Staphylococcus aureus* (MRSA)	Nanophotonic Interferometric Biosensor	49 and 29 CFU/mL was estimated for *P. aeruginosa* and MRSA	Whole cell	[116]
*Acinetobacter baumannii, Escherichia coli* and methicillin-resistant *Staphylococcus aureus* (MRSA)	Colorimetric Nitrocellulose membrane-based integrated microfluidic system	450 CFU	Whole cell	[117]
*Acinetobacter baumannii*	Fluorescence	100 CFU/reaction	Whole cell	[118]
*Acinetobacter baumannii*	Fluorescence	10 CFU/mL	Whole cell	[119]
*Campylobacter jejuni*	Fluorescence	2 CFU/mL	Whole cell	[109]
*Listeria monocytogenes*	Fluorescence	1–10 CFU/mL	Whole cell	[109]
*Listeria monocytogenes*	Electrochemical Amperometric	9–10^7^ CFU/mL	Whole cell	[120]
*Salmonella typhimurium, Bacillus subtilis, E. coli, and Listeria*	Electrochemical Amperometric	8 CFU/mL	Whole cell	[121]
*Salmonella*	Fluorescence polarization	a single bacterium	Whole cell	[122]
*Salmonella enterica*, *Escherichia coli* and *Listeria monocytogenes*	SpinChip Integrated with magnetic nanoparticles	10 CFU/mL	Whole cell	[123]
*Salmonella typhimurium and Saphylococcus aureus*	Gold nanoparticles Surface-Enhanced Raman Scattering	35 CFU/mL (*S. aureus*) 15 CFU/mL (*S. typhimurium*)	Whole cell	[124]
*Salmonella Enteritidis*	Allosteric Probe-Initiated Catalysis and CRISPR-Cas13a Amplification	1 CFU/mL	Whole cell	[125]
Listeriolysin O protein (Listeria)	Fluorescence	4–61 cells	Protein	[126]
Lipopolysaccharide	FRET	8.7 ng/mL	Lipoglycans	[127]
Lipopolysaccharide of *E. coli* 3 strains - ATCC 25922, DH5 alpha, and field isolate	Electrical Capacitance sensor	10^2^ cells/mL	Whole cell	[128]
Lipopolysaccharide from *Escherichia coli* 055:B5	FRET	7.9 fM (water) 8.3 fM (serum)	Lipoglycans	[129]
Lipopolysaccharide from *E. coli* O111:B4	Optical Colorimetric	1 ug/mL	Lipoglycans	[130]
Lipopolysaccharides from *E. coli*	Electrochemical Amperometric	29 ag/mL	Lipoglycans	[131]
Lipopolysaccharides from *Escherichia Coli*	Electrochemical Amperometric	1 fg/mL	Lipoglycans	[132]
Lipopolysaccharides	Optical Colorimetric	1.73 ng/mL	Lipoglycans	[133]
Lipopolysaccharides from *Salmonella entericaserotype typhimurium, Pseudomonas aeruginosa* 10 and *Escherichia coli* 055:B5	Fluorescence polarization	38.7, 88.0, and 154 ng/mL, respectively	Lipoglycans	[134]
*Bacillus anthracis* spore stimulant	Electrochemical Impedimetric	3 × 10^3^ CFU/mL	Spore	[135]
*Staphylococcus aureus* enterotoxin B	Fluorescence	0.9 pg/mL	Protein	[136]
*Staphylococcus aureus* enterotoxin B	Electrochemical Impedimetric	0.21 fM	Protein	[137]
*Staphylococcus aureus* enterotoxin B	Optical Colorimetric	50 ng/mL	Protein	[138]
*Staphylococcus aureus* enterotoxins	Fluorescence	1 ng/mL	Protein	[139]
*Streptococcus mutans*	Optical Colorimetric	12 CFU/mL	Whole cell	[140]
*Vibrio alginolyticus*	Electrochemical Amperometric	10 CFU/mL	Whole cell	[141]
*Vibrio parahemolyticus*	Optical Colorimetric	10 CFU/mL	Whole Cell	[142]
*Mycoplasma*	Surface Enhanced Raman Scattering	30 copies DNA/µL	Whole cell	[143]
*Staphylococcus aureus* protein A	Electrochemical Impedimetric	10 CFU/mL	Protein	[144]
*Staphylococcus aureus* protein A	Aptamer based optical silicon biosensor	3.17 µM	Protein	[145]

## Abbreviations

MRE	Molecular recognition elements
LOD	Limit of detection
MRSA	Methicillin-resistant Staphylococcus aureus
LPS	Lipopolysaccharides
SERS	Surface-enhanced Raman scattering
SPR	Surface plasmonresonance
FRET	Förster resonance energy transfer
SELEX	Systematic evolution of ligand by exponential enrichment
GO	Graphene oxide
AuNP	Gold nanoparticles
CFU	Colony forming unit
PCR	Polymerase chain reaction
